# Maternal and Fetal Outcomes of Motor Vehicle-Related Trauma in Pregnancy: A Trauma Center Study

**DOI:** 10.7759/cureus.99660

**Published:** 2025-12-19

**Authors:** Avis L Chan, Kristina Roloff, Michael Neeki, Jessica Sparks, Guillermo J Valenzuela

**Affiliations:** 1 Department of Obstetrics and Gynecology, Kaiser Permanente Santa Clara Medical Center, Santa Clara, USA; 2 Department of Obstetrics and Gynecology, Arrowhead Regional Medical Center, Colton, USA; 3 Department of Emergency Medicine, Arrowhead Regional Medical Center, Colton, USA; 4 Department of Emergency Medicine, California University of Science and Medicine, Colton, USA; 5 Department of Chemical, Paper, and Biomedical Engineering, Miami University, Oxford, USA

**Keywords:** abdominal trauma in pregnancy, airbag deployment, intra-abdominal injury, motor vehicle collision, obstetric trauma

## Abstract

Objective

The main objective of this study is to examine immediate outcomes, and the incidence of severe intra-abdominal injury after motor vehicle collisions (MVCs) in the pregnant population.

Methods

We conducted a retrospective cohort study. From 2015 to 2021, all pregnant patients who presented to a level I trauma center within 24 hours of MVC were included. Data on patient characteristics, collision details, hospital workup, and trauma-related injuries were extracted from their charts using ICD-10 code O9A.21. Descriptive statistics were used to summarize data. General linear models and univariate logistic regression models were used to analyze the relationship between variables.

Results

The study included 157 pregnant patients. Most were in their mid-20s, overweight to obese, multiparous, Hispanic, and in the second trimester. The median reported car speed was 30 mph (IQR 10-45 mph); 99% (N = 155) of patients reported use of seat belts, and 70% (N = 111) were drivers. Most collisions were hit from side. Airbags were deployed in 66% (N = 104) of collisions, and airbag deployment was significantly associated with a higher car speed (p < 0.01); 13% (N = 20) of patients had seat belt signs, and only 1% (N = 2) had peritoneal signs leading to exploratory laparotomy, while 85% (N = 133) of patients had no or minor injuries. Univariate logistic regression analysis showed car speed was significantly associated with intra-abdominal injury (p = 0.02). Car speed also independently predicted skin lacerations (p < 0.01) and length of hospital stay (p < 0.01), but not bony fractures (p = 0.15). Car speed did not vary with gestational age or maternal age. A trend was observed that pregnant individuals with increasing parity were involved in MVCs at lower car speeds.

Conclusion

In our study, a higher car speed is the main factor associated with increased injury severity, but not with the deployment of airbags. The majority of patients experienced no or only minor injuries, likely due to the high rate of seat belt use. Pregnant individuals with higher parity tended to be involved in crashes at lower speeds.

## Introduction

Motor vehicle collision (MVC) is a significant cause of trauma in pregnancy, leading to adverse maternal and fetal outcomes [1,2]. Three percent of pregnant individuals were involved in at least one MVC [3,4]. Maternal death occurred in 3.6 per 1,000, and fetal death or stillbirth in 6.76 per 1,000, based on a meta-analysis of 19 population-level studies [5]. The effects of blunt abdominal trauma can lead to a host of complications, including visceral organ injuries, bony fractures, placental abruption, preterm birth, low birth weight, exposure to multiple radiological examinations, and non-obstetric surgery [4-8]. Pregnant individuals are vulnerable to adverse consequences following MVC as a result of physiological, hemodynamic, and anatomical changes, which may hamper correct and timely diagnosis of visceral organ injury.

Much of the evidence on outcomes following MVC in pregnancy originates from national databases, such as the National Trauma Data Bank, National Automotive Sampling System/Crashworthiness Data System (NASS/CDS), Fatality Analysis Reporting System (FARS), Nationwide Inpatient Sample (NIS) database, and National Vital Statistics System (NVSS). Although these databases provide population-level data on over 10,000 subjects, they are prone to limitations in miscoding, over-generalization of diagnoses and conditions, and missing data [9]. Many studies appropriately conclude the need to study additional variables, such as collision characteristics, severity and type of trauma, car speed, occupant position, seat belt use, airbag deployment, demographics of occupants, and clinical data related to pregnancy, to better understand the relationship between MVC and injury outcomes in pregnant individuals [5].

This study, at a single trauma center, examines the immediate outcomes and the incidence of severe intra-abdominal injury, involving visceral organ laceration or rupture, after different types of MVC in the pregnant population.

## Materials and methods

We performed a retrospective observational cohort study using a convenience sample, from September 1, 2015, to December 31, 2021, at a level-one trauma center and the second-busiest Emergency Department in Southern California. This study was approved by the Institutional Review Board (protocol #22-25). Patient consent was waived for retrospective chart reviews.

To reduce misclassification bias, we extracted all cases at the institution with the World Health Organization ICD-10-CM diagnosis code O9A.21 (injury, poisoning, and certain other consequences of external causes complicating pregnancy) from the electronic medical record [10]. Patient charts were then reviewed by trained investigators, and the final study cohort included cases that met the following inclusion criteria: (1) pregnancy of any gestational age, and (2) presentation to the institute within 24 hours of MVC. Cases were excluded if the charts were significantly incomplete or if the type of documented trauma was not an MVC. In a few cases in which patients had more than one encounter following MVC, we only included the first encounter.

Maternal age, gestational age, gravidity, parity, body mass index on hospital admission, body height, body weight, race/ethnicity, and self-reported substance or alcohol use prior to the MVC were extracted from the medical records. Race/ethnicity, as self-reported data, was combined into five major groups: non-Hispanic White, Hispanic, non-Hispanic Black, Asian/Pacific Islander, and others. Car speed, use of a lap and shoulder seat belt, occupant’s position within the car (driver, front passenger, and rear passenger) at the time of collision, collision point on the car, car rollover, and deployment of airbags were reported and extracted during chart review. Significant findings included the presence or absence of a seat belt sign, peritoneal signs, serum alcohol or urine toxicology screening, ultrasound, computed tomography (CT) scan, abdominal surgery, injury details, and length of hospital stay.

Descriptive statistics were used for patient characteristics, collision characteristics, and maternal and fetal outcomes. Chi-squared test or Fisher's exact test was used, where appropriate, for categorical variables, while Student’s t-test was used for continuous variables. General linear models were performed to examine the relationship between two continuous variables. In addition, univariate logistic regression models were run to explore the association between intra-abdominal visceral organ injury and other patient or collision characteristics. Data were analyzed using SAS® version 9.3 (SAS Institute, Cary, NC, USA). A p-value less than 0.05 was considered statistically significant. 

## Results

**Figure 1:**
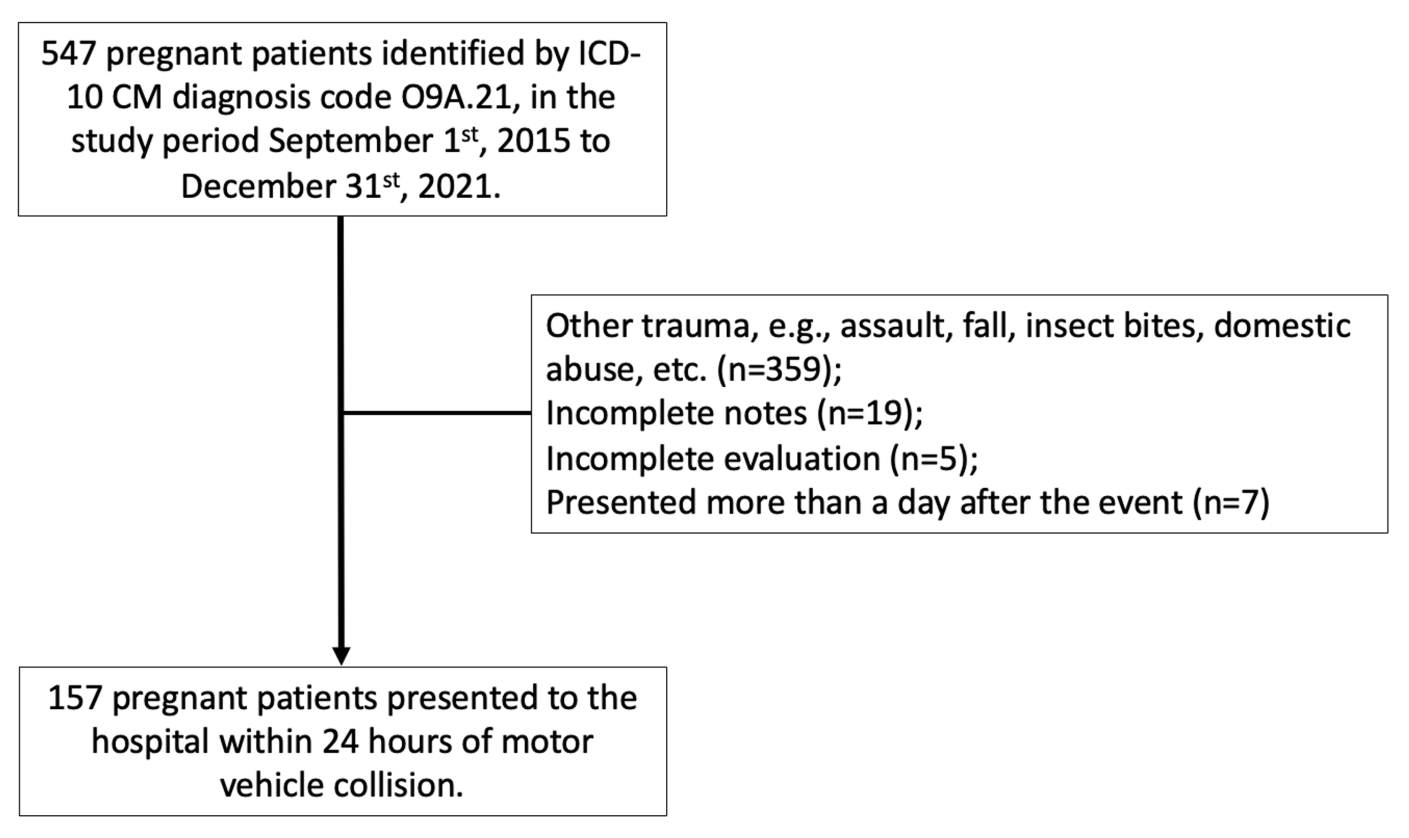
Inclusion and exclusion chart for the motor vehicle collision study in pregnant individuals World Health Organization: ICD-10-CM diagnosis code O9A.21 [10].

A total of 157 pregnant occupants were presented to the institute within 24 hours of an MVC for evaluation, from September 1, 2015, to December 31, 2021, in Figure 1.

Twenty-four patients were excluded due to incomplete documentation or incomplete evaluation. The majority of the patients were in their mid-20s, at least overweight to obese, with maternal height more than five feet, multiparous, Hispanic, and in their second trimester (Table 1). The median reported car speed was 30 mph (interquartile range (IQR) 10-45 mph). Of the total, 99% (N = 155) of patients reported use of seat belts, while 70% (N = 111) were drivers. Most collisions involved side impacts to the car. Only 5% (N = 8) of collisions involved rollover of the car, and there was no observed association between car rollover and speed (p = 0.1135). Airbags were deployed in 66% (N = 104) of the MVCs, and airbag deployment was statistically associated with car speed (p < 0.0001).

**Table 1:** Patient characteristics in the study cohort of pregnant individuals after motor vehicle collision Abbreviations: IQR, interquartile range; N, number; SD, standard deviation; CT, computed tomography

Patient characteristics	Value
Maternal age (years), mean ± SD	27.0 ± 5.8
Gestational age (weeks), mean ± SD	24.4 ± 8.7
Gravidity, median ± IQR	2.0 ± 1.0
Parity, median ± IQR	1.0 ± 2.0
BMI (kg/m^2^), mean ± SD	31.1 ± 7.1
Maternal height (inches), mean ± SD	63.9 ± 2.6
Race/ethnicity, N (%)	
Non-Hispanic White	17 (10.8)
Hispanic	117 (74.5)
Non-Hispanic Black	18 (11.5)
Asian/Pacific Islander	3 (1.9)
Others	2 (1.3)
Collision characteristics	
Speed (mph), median ± IQR	30.0 ± 35.0
Use of seat belt, N (%)	155/157 (98.7)
Occupant position at the time of collision, N (%)	
Front driver seat	111/157 (70.7)
Front passenger seat	41/157 (26.1)
Rear seat	5/157 (3.2)
Collision point on the car, N (%)	
Front of the car	34/157 (21.7)
Side of the car	70/157 (44.6)
Back of the car	50/157 (31.8)
Multiple areas	3/157 (1.9)
Car rollover, N (%)	8/157 (5.1)
Deployment of airbag, N (%)	104/157 (66.2)
Hospital workup	
Seat belt sign, N (%)	20/157 (12.7)
Peritoneal sign, N (%)	2/157 (1.3)
Stated history of substance/alcohol use, N (%)	4 (2.6)
Positive serum alcohol test, N (%)	5/107 (4.7)
Positive urine toxicology screening, N (%)	3/67 (4.4)
Ultrasound abdomen and pelvis, N (%)	138/157 (87.9)
CT abdomen and pelvis, N (%)	9/157 (5.7)

When patients presented to the hospital, 13% (N = 20) had seat belt signs, and only 1% (N = 2) had peritoneal signs, which led to exploratory laparotomy. Nearly 85% (N = 133) of patients had no or minor injuries (Table 2); 15% (N = 24) had skin lacerations; 4% (N = 6) had fractures; and 1% (N = 2) suffered intra-abdominal visceral organ injuries with hemoperitoneum.

**Table 2:** Maternal and fetal outcomes in the study cohort of pregnant individuals after motor vehicle collision Abbreviations: IQR, interquartile range; N, number

Maternal and fetal outcomes	Value
No obvious injury, N (%)	133 (84.7)
Skin lacerations, N (%)	24 (15.3)
Fractures, N (%)	6 (3.8)
Hemoperitoneum, N (%)	2 (1.3)
Intra-abdominal visceral organ injury, N (%)	2 (1.3)
Placental abruption, N (%)	1 (0.6)
Delivery at the admission episode, N (%)	1 (0.6)
Fetal demise, N (%)	1 (0.6)
Maternal death in hospital, N (%)	0
Length of hospital stay (days), median ± IQR	1.0 ± 1.0

The first of the two patients was 23 years old, gravida 2, para 1, eight weeks pregnant, and was brought in by ambulance after a head-on MVC at a speed of 90 mph. She was the restrained front-seat passenger. The airbag was deployed. She lost consciousness and suffered multiple extremity open fractures and lacerations. Hemoperitoneum was noted on focused assessment with sonography in trauma (FAST) and CT scans. An exploratory laparotomy was performed 33 minutes after arrival; findings revealed 150 mL of blood in the peritoneal cavity, a serosal tear over the cecum, a mesenteric hematoma, and hepatic lacerations on segments 4, 5, and 8. She subsequently had a miscarriage two days after surgery, which was managed medically.

The second patient with visceral organ injury was 27 years old, gravida 3, para 1, 18 weeks and one day pregnant, and was brought in after her car hit the center divider of a freeway at a speed of 70 mph and was then struck from the rear. She was a restrained front-seat passenger. The airbags were deployed. Free fluid was observed on abdominal ultrasound and CT. An exploratory laparotomy was performed 60 minutes after presentation, revealing 1500 mL of hemoperitoneum. A 5-cm fundal uterine laceration was noticed and repaired with hemostatic stitches. She eventually underwent emergency cesarean delivery for severe abdominal pain and suspected uterine rupture three months after the MVC. This case was reported in another article [11].

In addition to the two cases with visceral injury, we noted a patient with placental abruption after an MVC. She was 38 years old, gravida 2, para 1, 35 weeks and 6 days pregnant, and was brought in after a head-on car collision at a speed of 40 mph. She was a restrained driver. The airbags were not deployed. Severe hypertension was noted (maximum blood pressure 175/113), and she was admitted for preeclampsia with severe features. Six hours later, she developed heavy vaginal bleeding with recurrent late decelerations on continuous fetal heart monitoring. An emergency cesarean delivery was performed. During delivery, 10% of the placenta was found to have abrupted. It was unclear if the MVC, preeclampsia, or both caused the placental abruption.

There were no maternal deaths in this study. Since most patients had no or minor injuries, the median length of hospital stay was one day (IQR 1-2).

**Figure 2:**
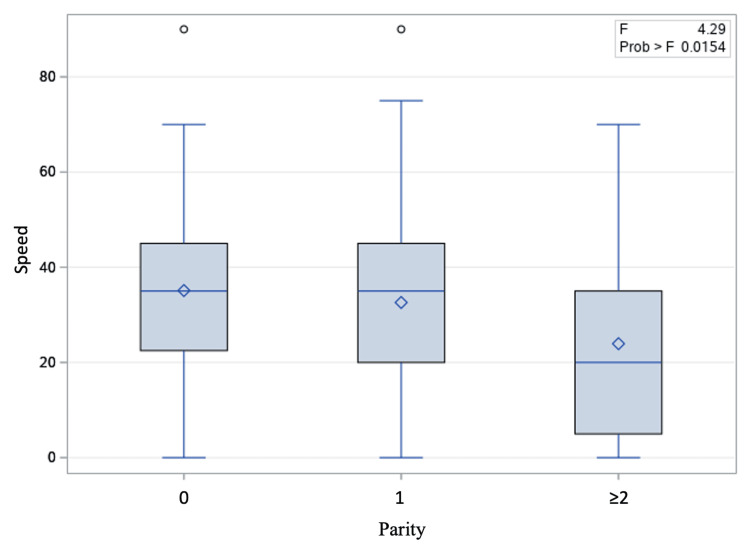
Box plots showing the relationship between parity and car speed in pregnant individuals involved in motor vehicle collisions

On univariate logistic regression, car speed was found to be significantly associated with intra-abdominal visceral organ injury (p = 0.0204). Car speed also independently predicted skin lacerations (p = 0.0001) and length of hospital stay (p < 0.0001), but not bony fractures (p = 0.1504). Car speed did not vary with gestational age or maternal age. However, there was a trend that pregnant individuals with parity of two or greater were involved in MVCs at lower car speeds (Figure 2). No associations or trends were observed for other factors, including gestational age and maternal age. Of note, we did not observe any differential preference for abdominal and pelvic imaging (CT scan or ultrasound) based on race/ethnicity after adjusting for car speed, gestational age, and maternal age (OR 1.667, 95% CI, 0.633-4.394, p = 0.3010).

## Discussion

Our retrospective cohort study provides additional information on patient and collision characteristics of MVCs involving pregnant individuals at a high-volume level I trauma center. In our study cohort, most pregnant patients were in their mid-20s and in the second or early third trimester. The racial distribution in our study cohort was comparable with that of the county population [12]. Our patients had very high self-reported seat belt use - up to 99% - which was much higher than the 73% reported by another academic institution in the proximal neighborhood [7]. Airbags were also deployed more frequently in our study cohort (66% vs. 6%) [7]. The differences in airbag deployment could be explained by the direction of force in the crash, car speed, car model, recall bias, and other factors. Due to insufficient data in both studies, direct comparison was not feasible. Our study also noted no or minor injuries in the majority of patients. Only 15% of patients had skin lacerations, open wounds, or bony fractures. Intra-abdominal visceral organ injury, placental abruption, immediate baby delivery, and fetal demise were uncommon (N = 3, 2%).

The study attempted to explore predictive factors for intra-abdominal visceral organ injury but found this difficult due to the low number of cases in the study cohort. We observed that intra-abdominal visceral organ injury was more likely with high car speeds (p = 0.0204, univariate logistic regression). This finding was consistent with another Japanese national study, which showed increasing odds ratios for moderate to severe injuries with higher car speeds (21-40 km/h: OR, 3.03; 41-60 km/h: OR, 13.47; ≥61 km/h: OR, 44.56) [13].

In the field of impact mechanics, a parameter termed delta V - the change in speed that a vehicle undergoes as a consequence of crashing - is widely used as an indicator of crash severity [14,15]. Klinich et al. analyzed crashes involving 57 pregnant occupants and found that higher crash severity, more severe maternal injury, and lack of proper seatbelt use were associated with an increased risk of adverse fetal outcomes [16]. Tanaka et al. recently developed a computer model of a pregnant occupant using a mathematical approach known as the finite element method to quantify the predicted area of placental abruption at different collision velocities [17].

It was reported that for simulations of unrestrained pregnant drivers, doubling the collision velocity increased the area of placental abruption tenfold. They also found that when the placenta was located at the uterine fundus, simulated steering wheel contact to the abdomen 30 mm above the umbilicus led to a smaller placental abruption area than contact at or 30 mm below the umbilicus. However, the authors noted that for simulations involving the higher contact location 30 mm above the umbilicus, deformations of the maternal abdominal organs may correspond to severe liver damage or rupture, possibly leading to fatal hemorrhage and fetal loss. Taken together, these findings support that lower collision speed and the use of properly positioned seatbelts are important to protect pregnant individuals and their fetuses in MVCs [16,17].

Controversies exist in the literature regarding airbag safety in maternal and fetal outcomes, especially because the distance between the gravid uterus and the airbag is usually less than 10 inches - the minimum distance recommended by the National Highway Traffic Safety Administration. A number of case reports were published in the 1990s about complications such as fetal skull and brain injuries and uterine rupture [18-21]. A state-level study in Washington concluded that there was no evidence of more adverse pregnancy outcomes with the deployment of airbags [22]. Our study also did not show any statistically significant increase in the risk of severe abdominal injury with airbag deployment, occupant seat position, or collision point on the car.

Many studies have investigated the modification in driving behaviors and habits during pregnancy. A study in North Carolina and a survey in France both found that pregnant individuals were at lower risk of being drivers in the last few weeks of gestation [23,24]. We did not notice this pattern in our study cohort, probably because only two patients presented at term pregnancy. However, we observed that the car speed involved in MVC was associated with parity, especially with two or more prior livebirths. This might reflect the experience of people driving more safely after having children [25]. Further research is needed to elaborate on driving habits and behavioral modification during pregnancy and the peripartum period. 

The strength of this study included a detailed chart review of 157 patients presenting in a busy level I trauma center in California. We included patient and collision characteristics that were not generally available in population-level datasets. This study, however, had several limitations. First, collision data were mostly self-reported, with some input from emergency medical services summoned to the scene. This type of data may be subject to recall bias. It is possible that pregnant occupants may avoid reporting violations against traffic law, such as speeding, lack of seat belt use, identity switch between drivers and passengers, or use of illicit drugs or alcohol. Second, we did not have information about seat belt types and car models. Differences in their features might impose differential mechanical impact on occupants, which could potentially lead to different extents of injuries. Third, 24 patients were excluded due to incomplete documentation or incomplete evaluation, which could lead to selection bias. These patients either left against medical advice or were asymptomatic after a minor MVC. Fourth, lack of follow-up after discharge might result in underestimation of maternal and fetal complications. Lastly, intra-abdominal visceral organ injury was uncommon, and, therefore, adjustment for confounding factors was impossible. Multi-center studies or a longer study period might be able to resolve this issue.

## Conclusions

MVCs are the most life-threatening type of trauma in pregnancy. Our study showed a very high usage of seat belts in pregnant individuals, a low proportion of airbag deployment, and no or minor injuries in most accidents, with only 1% leading to severe intra-abdominal injury.

A higher car speed was associated with intra-abdominal visceral organ injury, skin lacerations, and longer hospital stay, but not with the deployment of airbags. The parity of a pregnant woman may modify her driving habits, including reduced car speed. We did not observe any differential preference for abdominal and pelvic imaging (ultrasound and CT) based on race/ethnicity, after adjustment for car speed, maternal age, and gestational age. More studies involving multiple centers, a longer study period, and behavioral psychology are needed to better understand the effects of MVC on pregnant individuals and to identify other potential risk factors for severe intra-abdominal injury.
